# Experimental Study on Shear Behavior of Non-Stirrup Ultra-High Performance Concrete Beams

**DOI:** 10.3390/ma16114177

**Published:** 2023-06-04

**Authors:** Pingjie Li, Quan Cheng, Nanxun Chen, Yueqiang Tian, Junfa Fang, Haibo Jiang

**Affiliations:** 1 School of Civil and Transportation Engineering, Guangdong University of Technology, Guangzhou 510006, China; 2112109004@mail2.gdut.edu.cn (P.L.); 3117003038@mail2.gdut.edu.cn (Q.C.); 2 Guangdong Zhonglu Protection Technology Co., Ltd., Guangzhou 511430, China; chennanxun2023@126.com; 3 Zhonglu Xincai (Guangzhou) Technology Co., Ltd., Guangzhou 511430, China; tian_t4@126.com; 4 Zhonglu Dura International Engineering Co., Ltd., Guangzhou 510430, China; fangjunfa2023@126.com

**Keywords:** shear behavior, ultra-high performance concrete (UHPC), non-stirrup UHPC beams, steel fiber, shear span-to-depth ratio

## Abstract

Due to the high tensile strength of ultra-high performance concrete (UHPC), the shear stirrups in UHPC beams could potentially be removed. The aim of this study is to assess the shear performance of non-stirrup UHPC beams. Six UHPC beams were tested and compared with three stirrup-reinforced normal concrete (NC) beams, taking into consideration the testing parameters of steel fiber volume content and shear span-to-depth ratio. The findings demonstrated that incorporating steel fibers can efficiently strengthen the ductility, cracking strength, and shear strength of non-stirrup UHPC beams and alter their failure mode. Additionally, the shear span-to-depth ratio had a significant impact on the shear strength of beams, as it was negatively related to it. This study revealed that the French Standard and PCI-2021 formulae were suitable for designing UHPC beams with 2% steel fibers and no stirrups. When applying Xu’s formulae for non-stirrup UHPC beams, taking into account a reduction factor was necessary.

## 1. Introduction

UHPC is a new-generation composite material made of cement, mineral additions, fine aggregates, steel fibers, water reducer, and potable water. With extraordinary mechanical properties, including excellent ductility, durability, tensile and compressive strengths [1,2,3,4,5], UHPC is considered a game-changing innovative engineering material [6]. In addition, UHPC has a certain self-healing ability, which can effectively resist the corrosion of steel reinforcement within the structure by aggressive waters [7,8]. Hence, UHPC is popularly used in engineering applications, particularly in bridge engineering. Prominent examples of its application in bridge engineering include the Sherbrooke Pedestrian Bridge in Canada, the world’s first prestressed UHPC structure bridge, the first UHPC highway bridge in the United States, the Mars Hill Bridge, completed in Iowa in 2006, the first prestressing UHPC girder bridge without stirrups in China built by Zhonglu Dura Co., Ltd. in China in 2018, the Guangzhou North Ring Expressway Expansion F Ramp Bridge. There are also many studies on the bridge aspect of UHPC, including steel-UHPC composite girders [9,10,11,12,13], prefabricated segmental girders [14], and joints [15,16,17].

In normal concrete beam structures, dense stirrups are commonly arranged to resist shear forces. Stirrups play a critical role in altering the shear failure mode of a structure from a brittle to a ductile type by improving shear capacity and enhancing the ductility of normal concrete (NC) beams [18,19,20]. The number of stirrups intersecting the critical diagonal crack, along with the diameter and yielding strength of the stirrups, are key parameters impacting the contribution of stirrups to the shear capacity provided by the beam [21]. However, some of the stirrups crossing the critical diagonal crack did not attain the yield strength at the beginning of the peak shear resistance [21].

Adding the right quantity of steel fibers to the UHPC matrix can considerably increase its tensile strength [22,23]. Steel fibers can act like stirrups by directly bridging diagonal cracks or indirectly transferring stresses to both sides of the crack, redistributing the stress distribution. Additionally, steel fibers have multiple benefits, such as enhancing the post-cracking shear behavior of concrete [24], leading to better and smaller distributed cracks in concrete members [25], improving fatigue life without stress reversals [26], and also increase the flexural and shear capacity [27]. The orientation, distribution, shape, size, and volume of steel fibers are important factors that affect the performance of concrete [27,28,29,30]. It is important to note that steel fibers’ incorporation at a 0.5% volume content cannot completely replace stirrups to resist shear forces [31]. Increasing the volume content of steel fibers can make the shear strength of the beam improved to a large extent. However, when the volume content of steel fibers surpasses 3.0%, the mechanical properties of UHPC do not experience significant improvements, probably because of the agglomeration of steel fibers, which affected the flowability [32]. Therefore, the range of steel fiber commonly used in practical engineering is around 2.0–3.0%.

In general, steel fibers have a similar effect as stirrups and make up for the relatively low tensile strength of normal concrete. By making the most of the excellent tensile and compressive properties of UHPC, it is possible to create slender and more lightweight structural components. Substituting steel fibers for stirrups in beams can reduce the congestion of reinforcements in concrete beams, resulting in shorter construction time and lower costs while not hindering UHPC flowability [33]. The adequacy of steel fibers as a substitute for stirrups in providing sufficient shear resistance continues to be a subject of inquiry, which has spurred numerous researchers to explore the shear behavior of beams strengthened with steel fibers instead of traditional stirrups. The topic of eliminating stirrups in structural elements is a relatively new area of study, with research being conducted from 2010 up to today. There have been limited studies reported on this topic, as noted by Yulin Xiao [34] from the University of Florida in his 2014 investigation titled “Quantifying Ultra-High Performance Concrete Flexural System Mechanical Response”. Some recent studies on the topic include those conducted by AM Jabbar [35] in 2023 and J Yang [36] in 2022. Current research on non-stirrup UHPC beam shear performance largely concentrates on prestressing and thin-web I-sections [37,38,39,40]. UHPC I-girders without stirrups demonstrated good ductility, and the shear strength still increased gradually after initial cracking [37]. The quantity and kind of fibers used in non-stirrup reactive powdered concrete I-section girders did not significantly impact the cracking load; instead, they greatly influenced the failure load and expansion rate of cracks [38]. Raising the volume content of steel fibers can lead to higher crack strength and shear strength for externally non-stirrup prestressed precast UHPC segmental beams [39]. Concurrently, in non-stirrup prestressed UHPC I-section beams, shear cracking in the web was found to be distributed significantly before the dominant failure crack formed [40]. Moreover, scholars have conducted studies on various factors that can influence the shear capacity of non-stirrup UHPC beams, such as reinforcement ratio, steel fiber volume ratio, and the shear span-to-depth ratio [41,42,43,44,45]. The shear cracking of UHPC beams was influenced by factors such as cross-sectional shape, prestressing, and fiber orientation, in addition to the tensile behavior of the UHPC [46]. UHPC beams exhibited an average shear capacity approximately 3.5 times superior to that of NC beams, and the reinforcement rate had a minimal impact on the shear capacity of UHPC beams [47]. Nut anchoring for longitudinal reinforcement resulted in reduced shear capacity but increased ductility in NC beams, while this technique did not seem to have a significant influence on the shear capacity of UHPC beams [47]. Numerical simulations have proven to be a successful method used by numerous scholars to evaluate and predict the shear strength of UHPC beams [39,48,49].

Prestressing tendons have the capability to raise the shear capacity and span capacity and narrow the web thickness of non-stirrup UHPC beams [50]. However, for small span bridges, it is most common to eliminate the prestressing tendons due to the challenging nature of their construction, high cost, and difficulty in controlling the reverse arch. In this paper, three rectangular section UHPC beams with 2% steel fibers and without stirrups and three rectangular section UHPC beams without steel fibers and stirrups were designed and compared with three stirrup-reinforced NC beams. None of these specimens were reinforced with prestressing. The key test parameters included the presence or absence of steel fibers and shear span-to-depth ratio. The tested beams were analyzed by observing their failure modes, crack patterns, deformation, and ultimate shear strength. Furthermore, the accuracy of the French standard formula, PCI-2021 formula, and Xu’s formula were verified in this study. The research presented in this paper aims to be a significant contribution towards a deeper and more widespread understanding of non-stirrup UHPC beams in shear resistance.

## 2. Experimental Program

### 2.1. Specimen Description

**Figure 1:**
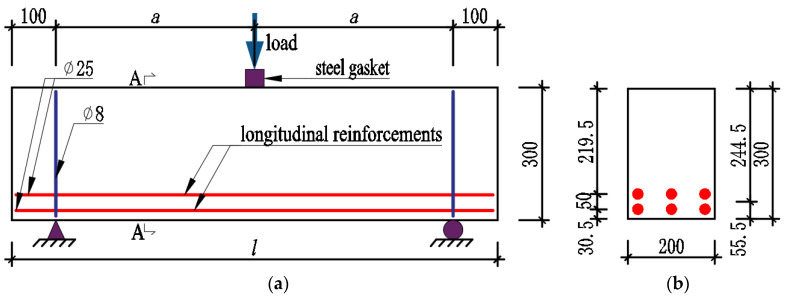

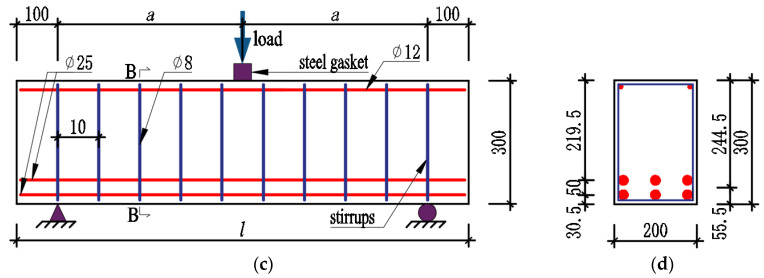
Dimensions and layouts of tested beams (unit: mm). (**a**) NSR−UHPC−0 beams and NSR−UHPC−2 beams; (**b**) Section A-A; (**c**) SR−NC beams; (**d**) Section B-B.

**Figure 2:**
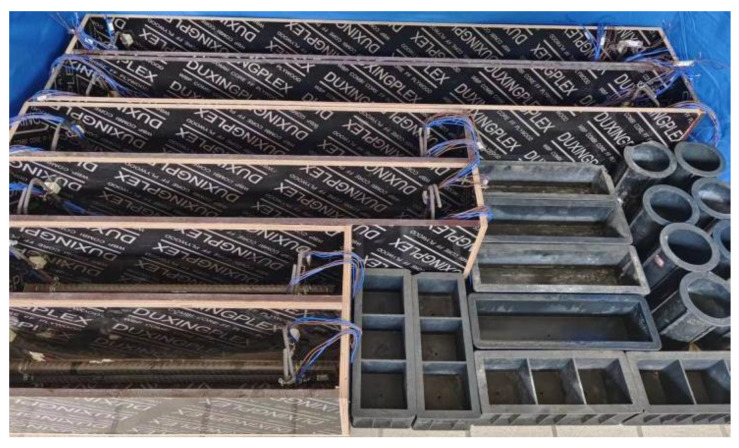
Formworks of NSR−UHPC−0 beams and NSR−UHPC−2 beams.

Nine beams were designed in this study, including six non-stirrup UHPC beams and three stirrup-reinforced NC beams as a comparison. The beam dimensions shown in Figure 1 and Figure 2 showcase the formworks of non-stirrup UHPC beams. The cross-section of the tested specimen was rectangular, with a uniform dimension of 300 mm×200 mm (h×b), where h was the overall height of the beam and b was the beam width. Three beam lengths were chosen: 800 mm, 1100 mm, and 1700 mm, each with a corresponding shear span-to-depth ratio of 1.2, 1.8, and 3.1, respectively. The shear span-to-depth ratio is calculated by the equation: λ=a/d. Here, the letters *a* and *d* indicated the length of the shear span and the effective depth of the beam, respectively. Additionally, in the following expressions, the shear span-to-depth ratio is represented by the symbol λ. Namely, *d* took the value of 244.5 mm, and the concrete cover was 10 mm. In order to cause shear failure, ribbed reinforcements were used with a 25 mm diameter and 425.1 MPa yield strength ($f_{y}$), and dual layers of longitudinal reinforcements were installed in the beams. The NC beams that employed double-leg stirrups utilized ribbed reinforcements with an 8 mm diameter arranged at 100 mm intervals.

As depicted in Table 1, the experimental variables include concrete type, with stirrups or without stirrups, volume content of steel fiber, and shear span-to-depth ratio. To clearly indicate the experimental variables in an organized manner, the specimens were identified with the nomenclature of C-S*-R*-V* or U-S*-R*-V*. The letters “C” and “U” represented the type of concrete used, with “C” indicating normal concrete and “U” indicating ultra-high performance concrete. The letters “S1” and “S0” specified whether the beams were constructed with stirrups or without stirrups, respectively. The numerical value succeeding the letter “R” denoted the shear span-to-depth ratio in the range of 1.2 to 3.1. Moreover, the letters “V2” and “V0” represented the volume content of steel fiber of 2% and 0%, respectively. All specimens were divided into three series, SR−NC beams, NSR−UHPC−0 beams, and NSR−UHPC−2 beams, where SR−NC beams represented stirrups reinforced NC beams, NSR−UHPC−0 beams represented non-stirrup reinforced UHPC beams without steel fibers, and NSR−UHPC−2 beams represented non-stirrup reinforced UHPC beams with 2% steel fibers.

### 2.2. Test Process and Instrumentation

**Figure 3:**
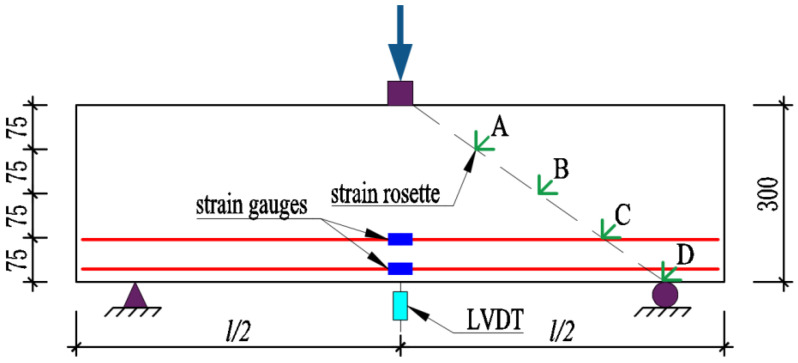
Layout of test devices (unit: mm).

**Figure 4:**
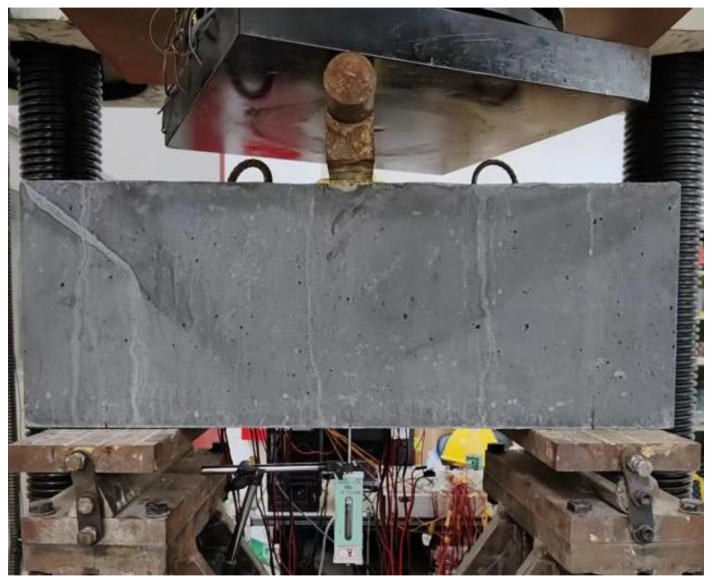
Loading configuration.

As shown in Figure 3 and Figure 4, with a view to evaluating the shear behavior of beams, three-point bending tests were conducted. An electric hydraulic machine with a capacity of 10,000 kN was employed to impose a concentrated load. Each beam was supported by a simple support system consisting of pin and roller supports placed at a distance of 100 mm from each end.

The deflections of the beams were monitored with the use of a Linear Vertical Displacement Transducer (LVDT) positioned at the mid-span of each beam. Four groups of strain rosettes marked A to D from top to bottom, were evenly arranged on the line extending from the loading point to the support. Each strain rosette group consisted of three strain gauges set at 0°, 45°, and 90° to the longitudinal direction, employed to measure the principal strains. The mid-span strains of the longitudinal reinforcements were recorded through strain gauges. The strain gauges used to measure the strains in the longitudinal reinforcements were adhered to the steel rebars. At the location of the paste, the steel rebars were sanded smooth to facilitate the application of strain gauges. After the strain gauges are attached, the strain gauges are protected by applying waterproof adhesive and anti-collision adhesive to the strain gauges. In general, the experimental results were adopted using devices with pressure transducers, strain gauges, and LVDT, all connected to the JMTEST static collector, which was used to collect the load force, longitudinal reinforcement strain, concrete strain, and mid-span deflection during the loading process. Additional information regarding the locations of strain gauges and LVDT can be seen in Figure 3.

Prior to the formal loading of the test, a 40 kN preload was applied to verify the proper connection and functionality of the loading devices and data acquisition instruments. Afterward, the preload was removed, and a gradual monotonic load was applied at 0.3 mm/min until shear diagonal cracks manifested. The loading rate was then reduced to 0.1 mm/min for the beam to incur significant damage, and the test was terminated.

### 2.3. Material Properties

**Figure 5:**
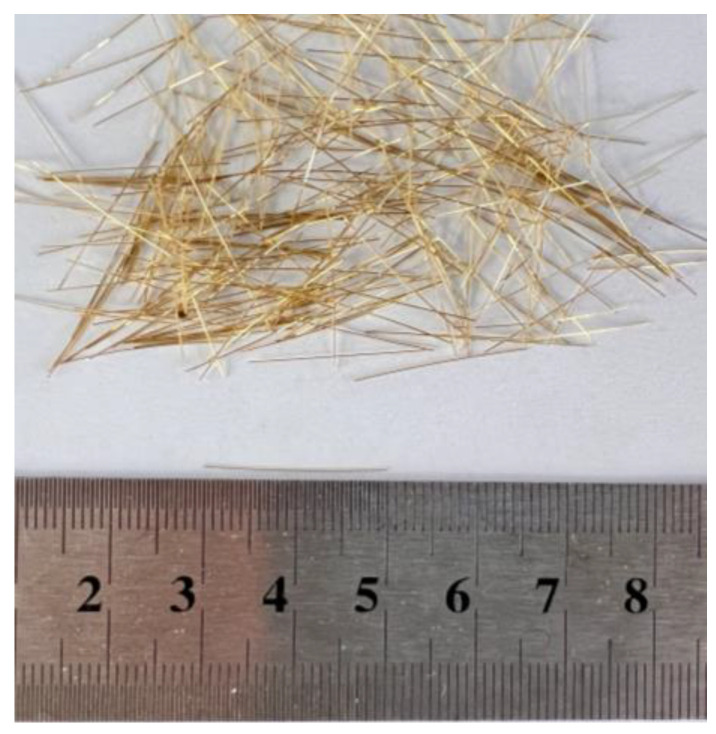
Type of steel fibers (unit: cm).

As shown in Table 2, the UHPC mixture mainly consists of the following components: quartz sand, silica fume, cement, Nano-CaCO_3_, steel fiber, water reducer, and water. The steel fibers used in the experiment had an equivalent diameter of 0.2 mm and a length of 20.0 mm, and their tensile strength was 2950 MPa, as shown in Figure 5. Two UHPC mixtures were examined with varying steel fiber volume contents: 0.0% and 2.0%. The 0.0% steel fiber volume content was utilized to analyze the contribution of steel fibers on the shear load capacities of beams. The 2.0% steel fiber volume content is a typical amount used in UHPC mixtures.

In accordance with French Standard NF P 18-470 [51] and French Standard NF P 18-710 [52], the cube compressive strength $f_{cu}$ was obtained utilizing the cubic samples with a side length of 100 mm, and the tensile performance of UHPC was determined by the prismatic bending test with a length of 100 mm, a width of 100 mm, and a height of 400 mm. The experimental post-cracking strength was obtained using the following equation:

(1) $$\sigma_{f1}=\frac{1}{w^{*}}\int_{0}^{w^{*}}\sigma_{f}\left(w\right)dw$$

The design value for post-cracking strength is expressed as follows:

(2) $$\sigma_{f2}=\frac{1}{K\gamma_{\mathrm{cf}}}\frac{1}{w^{*}}\int_{0}^{w^{*}}\sigma_{f}\left(w\right)dw$$

where the post-cracking stress $\sigma_{f}\left(w\right)$ is determined based on the crack opening w. The maximum crack width $w^{*}$ was chosen to be 0.3 mm. The fiber orientation factor *K* used in this study was considered to be 1.25, and the partial safety factor ($\gamma_{\mathrm{cf}}$) was chosen as 1.3; both values were taken using the recommended values of the French standard NF P 18-470.

In accordance with the PCI-2021 report [53], the residual tensile strength $f_{\mathrm{rr}}$ is determined as the first peak cracking value, as calculated by the following equation:

(3) $$f_{\mathrm{rr}}=\psi f_{\mathrm{fu}}$$

where ψ is a conversion factor from ultimate flexural strength $f_{\mathrm{fu}}$ to post-cracking tensile strength, which is taken as 0.375 in this paper.

According to Xu’s formula [54], the axial compressive strength $f_{c}$ was obtained by 300 mm×100 mm×100 mm prisms. Table 3 contains additional information on the properties of the concrete materials used.

Table 4 contains the test results of the mechanical properties of the stirrups and longitudinal reinforcements, which were evaluated using an electro-hydraulic servo machine.

## 3. Experimental Results and Discussion

### 3.1. Failure Modes and Crack Patterns

**Figure 6:**
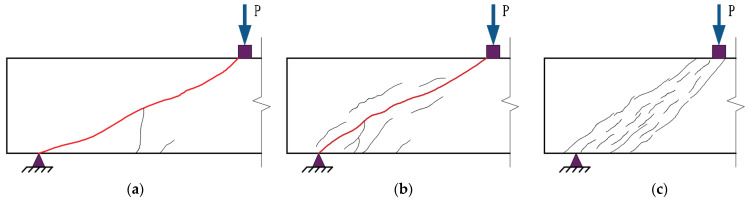
Types of shear failure. (**a**) Diagonal tension failure; (**b**) Shear compression failure; (**c**) Diagonal compression failure.

**Figure 7:**
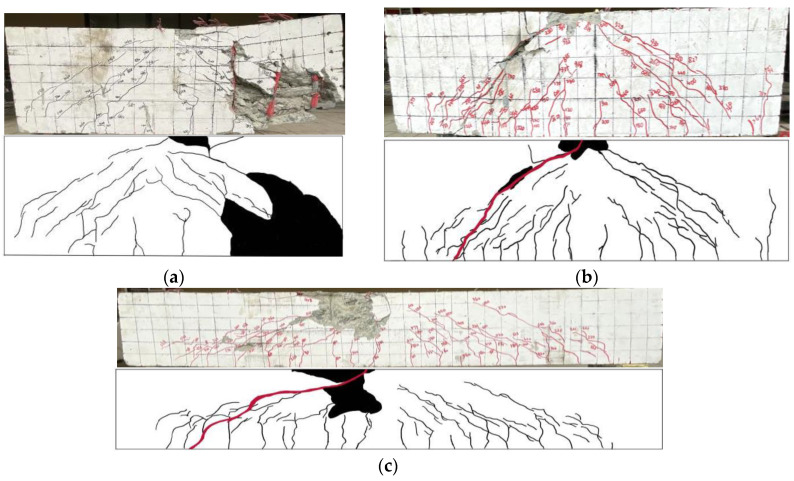

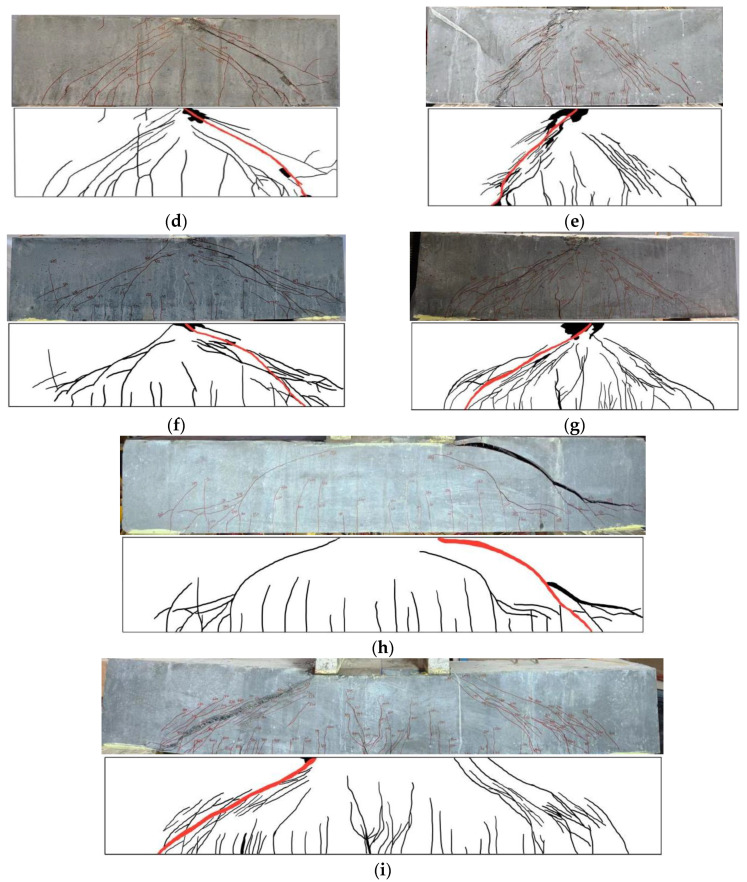
Crack diagram of each test beam. (**a**) B1; (**b**) B2; (**c**) B3; (**d**) B4; (**e**) B7; (**f**) B5; (**g**) B8; (**h**) B6; (**i**) B9.

Each of the beams tested demonstrated shear failures, specifically diagonal tension failure (DT), shear compression failure (SC), and diagonal compression failure (DC), which were identified as the typical types of shear failures, as shown in Figure 6. The different failure modes and crack patterns observed in all tested specimens are depicted in Figure 7. The critical cracks, which were identified at the end of the test, were visually represented by thick red lines, while the areas of severe concrete damage were denoted by black bolded portions.

Regarding specimen B1 (Figure 7a), few cracks were visible until a loading of 400 kN was applied, with bending cracks appearing near the middle of the span. Upon increasing the load, diagonal cracks started to develop and grow in size. After reaching the ultimate load of 1146 kN, specimen B1 was suddenly damaged, and the concrete of the support was crushed and spalled off. It can be inferred that the shear failure of specimen B1 was diagonal compression damage.

As depicted in Figure 7b–g,i, typical patterns of shear compression failures were illustrated. Initially, flexural cracks appeared at the bottom of the beam close to the point of load application during testing. Once the load reached approximately 30~50% of the ultimate load, the number of flexural cracks no longer increased, and the existing flexural cracks within the shear-bending section expanded diagonally toward the loading point. At the same time, new diagonal cracks kept appearing and intersecting with existing diagonal cracks to form small, short concrete columns, which gradually developed into a critical diagonal crack. Following the attainment of the ultimate load, concrete crushing was evidenced within the compression zone close to the point of loading.

Regarding specimen B6 (Figure 7h), its failure mode was classified as diagonal tensile failure. Once diagonal cracks surfaced within the shear-bending section, the crack width swiftly increased, and the cracks were pulled apart and developed into critical diagonal cracks soon. Finally, specimen B6 suddenly lost its load-bearing capacity and was torn into two parts with flat failure surfaces without any concrete crushing. More detail about the failure modes and test results can be found in Table 5.

#### 3.1.1. Effect of Shear Span-to-Depth Ratio on Failure Modes and Crack Patterns

In SR−NC beams, specimen B1 exhibited diagonal compression failure, while specimens B2 and B3 both demonstrated shear compression failure. The experimental results indicated that SR−NC beams, having lower λ, exhibited a significant improvement in their ability to withstand shear forces. However, due to the material’s abrupt failure, it may not be suitable for practical engineering applications.

Both UHPC beams with the λ of 1.2 and 1.8 failed due to shear compression. However, upon increasing the λ to 3.1, specimen B6 experienced diagonal tension failure, while specimen B9 still exhibited shear compression failure. It can be inferred that the failure mode of NSR−UHPC−2 beams appeared to be minimally affected by the λ of 1.2, 1.8, and 3.1. In contrast, as the shear span-to-depth ratio increased, the principal tensile stress soon exceeded the threshold tensile stress, and critical diagonal cracks then occurred in the shear-bending section of the NSR−UHPC−0 beam. This ultimately led to the failure of specimen B6 due to diagonal tension failure.

Table 5 lists the load of the initial flexural crack ($P_{\mathrm{cr}}$) and the corresponding cracking strength ($\sigma_{\mathrm{cr}}$) values. As the λ increased, there was a decrease in the loads of the flexural cracks observed in UHPC beams. However, the corresponding cracking strength did not exhibit significant differences. The coefficient of variation for the flexural cracking strength was low, with 0.02 for NSR−UHPC−0 beams and 0.10 for NSR−UHPC−2 beams. On the other hand, the flexural cracking strength exhibited significant variation in SR−NC beams. Specifically, while specimen B1 showed a high cracking strength of 10.0 MPa, specimens B2 and B3 displayed lower strengths of 5.3 MPa and 6.0 MPa, respectively. The coefficient of variation was 0.29. It can be inferred that the load of flexural crack decreased as the λ increased, but the impact of λ on the flexural cracking strength of NSR−UHPC−0 beams and NSR−UHPC−2 beams was insignificant, which mainly depended on the properties of the UHPC matrix.

Table 5 presents the load of the initial diagonal crack ($P_{\mathrm{ci}}$) and the corresponding cracking strength ($\nu_{ci}$). The diagonal cracking strength diminished as the λ increased. As the λ increased from 1.2 to 1.8 and from 1.8 to 3.1, the reduction in diagonal cracking strength was more pronounced for SR−NC beams (about 30%) and NSR−UHPC−0 beams (about 20%) compared to NSR−UHPC−2 beams (only about 6%). This implied that the NSR−UHPC−2 beam had a relatively smaller decrease in its diagonal cracking strength with the increase in the λ in comparison to the SR−NC beams and NSR−UHPC−0 beams.

#### 3.1.2. Effect of Steel Fibers on Failure Modes and Crack Patterns

As shown in Figure 7, steel fibers can influence the failure modes of the non-stirrup UHPC beams. For instance, by comparing specimens B6 and B9 with the identical λ, specimen B6 lacked the bridging action of steel fibers and quickly lost its load-bearing capacity. Nevertheless, reinforcing with steel fibers in specimen B9 contributed to its ability to maintain good ductility. Consequently, steel fibers effectively enhanced the safety of non-stirrup UHPC beams.

As shown in Table 5, steel fibers can suppress the appearance of cracks in beams, including flexural cracks and diagonal cracks. In NSR−UHPC−2 beams, the flexural cracking strength and diagonal cracking strength could be increased by up to 131.7% and 122.7%, respectively, compared with SR-C40 beams. Relative to NSR−UHPC−0 beams, steel fiber incorporation showed a significant enhancement in flexural cracking strength (up to 139.7%) and diagonal cracking strength (up to 145.0%). Based on these results, we can conclude that steel fibers were beneficial in delaying the appearance of cracks by improving the cracking strength.

### 3.2. Load-Displacement Relationships

**Figure 8:**
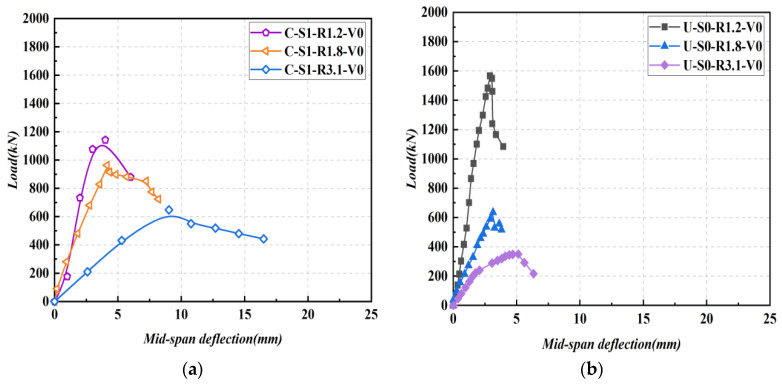

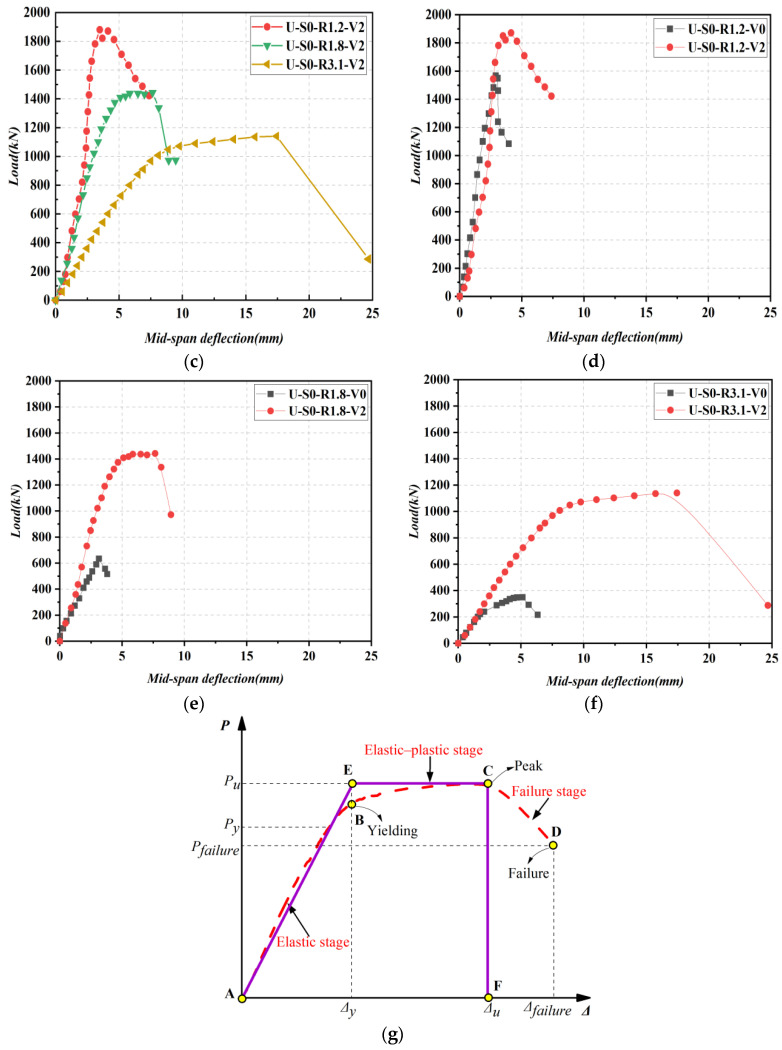
Load-mid span deflection curves. (**a**) Effect of λ of SR−NC beams; (**b**) Effect of λ of NSR−UHPC−0 beams; (**c**) Effect of λ of NSR−UHPC−2 beams; (**d**) Effect of steel fibers at a λ of 1.2; (**e**) Effect of steel fibers at a λ of 1.8; (**f**) Effect of steel fibers at a λ of 3.1; (**g**) Generalized P–Δ curve.

Figure 8 illustrates the load-displacement curves for all tested beams, with Figure 8a–f displaying the outcomes for various beam specimens that have different steel fiber volume contents and shear span-to-depth ratios. These results are summarized in load-mid span deflection curves (P–Δ curves) presented in Figure 8g. The ductility coefficient is a significant indicator that reflects the deformation behavior of the beam and is linked to the deflections at the yield point and the failure load (80% of the ultimate load). The main purpose of ductility is to prevent brittle damage to the structure, which is related to the number of longitudinal reinforcements, the dimensions of the cross-section, and the rotational capacity [55,56]. By utilizing the energy method, the yield point of the beam was determined by equating the integral area of curve AC with the area of a right-angle trapezoid (AECF). The displacement of the yield point was the length of AF minus the length of EC. The load and corresponding deflection at the yield point ($P_{y}$ and $\Delta_{y}$), at the ultimate load ($P_{u}$ and $\Delta_{u}$), and at the failure load ($P_{failure}$ and $\Delta_{failure}$), as well as the ductility coefficients ($\mu_{\Delta }$) are listed in Table 5.

Figure 8g exhibits the P–Δ curves that can be categorized into elastic stage, elastic-plastic stage, and failure stage. The slope of the curve in the elastic stage (curve AB) remained essentially constant. The load exceeded the yield point at point B as a result of the stirrups or longitudinal reinforcements yielding or the concrete crushing in the shear-bending section. The elastic-plastic stage (curve BC) showed a nonlinear increase in load-displacement due to material nonlinearity. As the loading increased, the elastic-plastic stage maintained a consistently small slope, eventually reaching the ultimate load at point C. This may be attributed to the enhanced strength of the longitudinal reinforcements. As the beam underwent the failure stage (curve CD), its load-bearing capacity decreased due to the crushing or separation of the concrete in the shear-bending section. As a result, various failure modes were observed in this stage.

As presented in Figure 8a–c, the stiffness of the beams was heavily influenced by the λ, which notably affected the slope of the elastic stage. With an increasing λ, the stiffness of the beams decreased while deflection increased, leading to the expansion of flexural crack. The impact of steel fibers on the P–Δ curve is shown in Figure 8d–f. At the beginning of the test, the impact of steel fibers was insignificant as the curves of the UHPC beams at this time had similar slopes. However, the trends of the elastic-plastic stage differ significantly. For NSR−UHPC−0 beams, their longitudinal reinforcement did not yield during the elastic-plastic stage, suggesting that the concrete bore most of the load, so the curve BC was extremely short. Notably, the NSR−UHPC−0 beams with a relatively small shear span depth showed this trend more clearly, with only a 40–50 kN increase from yield load to ultimate load and a small change in deflection. In contrast, the longitudinal reinforcements of NSR−UHPC−2 beams yielded and gradually strengthened between the curve BC, and the steel fibers were partially pulled out. This observation indicated that the longitudinal reinforcement, steel fibers, and concrete shared the load, resulting in an increase of approximately 95 kN from the yield load to the ultimate load with greater deflection. The ductility of NSR−UHPC−2 beams was significantly increased.

As shown in Figure 8d, the P–Δ curves of specimens B4 and B7 exhibited similar trends in the three stages. At a small λ of 1.2, the steel fibers in the non-stirrup UHPC beams did not play a significant role but only enhanced the ultimate load and deflection to a small extent. As illustrated in Figure 8e,f, the P–Δ curves of specimens B8 and B9 differed significantly from those of specimens B5 and B6. Non-stirrup UHPC beams with the λ of 1.8 and 3.1 were greatly impacted by the inclusion of steel fibers, resulting in a significant rise in ultimate load and deflection.

### 3.3. Strain Response

#### 3.3.1. Strain Response of Longitudinal Reinforcements

**Figure 9:**
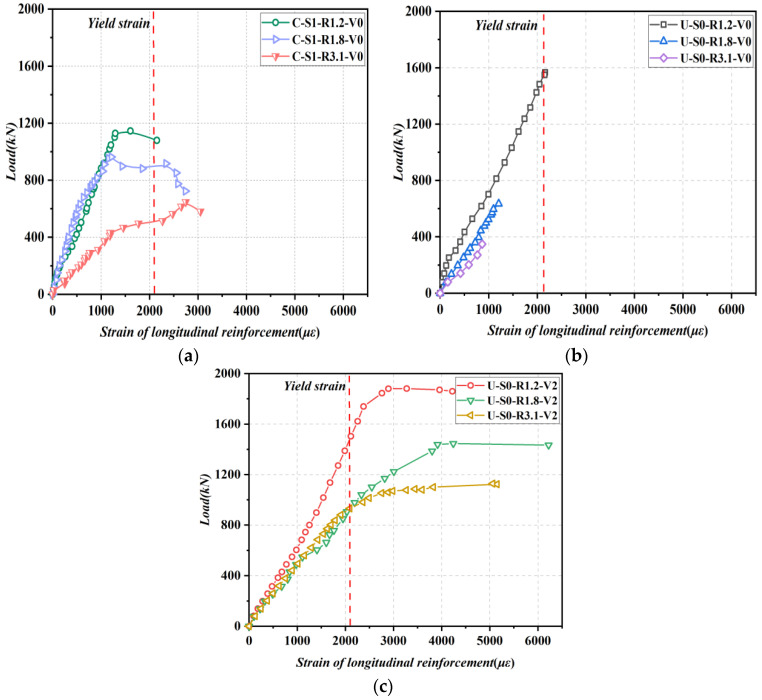
Load-strain curves of longitudinal reinforcements. (**a**) SR−NC beams; (**b**) NSR−UHPC−0 beams; (**c**) NSR−UHPC−2 beams.

Figure 9a–c shows the load-strain relationships of longitudinal reinforcements for SR−NC beams, NSR−UHPC−0 beams, and NSR−UHPC−2 beams, respectively. The tested beams were all made of longitudinal reinforcements with an average yield strength of 425.1 MPa and a modulus of elasticity of 2.0 × 10^5^ MPa, so the yield strain of the longitudinal reinforcements was about 2125.5 με. As presented in Figure 9a, the longitudinal reinforcement strains for specimens B1 and B2 reached the yield strain upon achieving the ultimate load, whereas specimen B3 had already yielded before the ultimate load was achieved. In Figure 9b, the longitudinal reinforcements of specimens B5 and B6 were far from yielding at the time of failure, while the longitudinal reinforcements of specimen B4 reached yield at the time of failure. In Figure 9c, the longitudinal reinforcements of specimens B7, B8, and B9 had all yielded before reaching the ultimate load. From these findings, it can be assumed that the steel fibers significantly enhanced the strength of the UHPC matrix, and the longitudinal reinforcements in NSR−UHPC−2 beams took more load, thus increasing the shear resistance of NSR−UHPC−2 beams.

#### 3.3.2. Strain Response of Concrete Diagonal Sections

Each strain rosette group consisted of three strain gauges set at 0° ($\varepsilon_{x}$), 45° ($\varepsilon_{45{}^{\circ}}$), and 90° ($\varepsilon_{y}$) to the longitudinal direction, which were used to calculate the principal tensile strains $\varepsilon_{t}$ and principal compression strains $\varepsilon_{c}$ by Equation (4). When the calculation result is positive, the strain is the principal tensile strain, and vice versa for the principal compressive strain.

(4) $$\left\{\begin{array}{c} \varepsilon_{t}\\ \varepsilon_{c} \end{array}\right.=\frac{\varepsilon_{x}+\varepsilon_{y}}{2}\pm \sqrt{{\left(\frac{\varepsilon_{x}-\varepsilon_{y}}{2}\right)}^{2}+{\left(\frac{\varepsilon_{x}+\varepsilon_{y}}{2}-\varepsilon_{45{}^{\circ}}\right)}^{2}}$$

**Figure 10:**
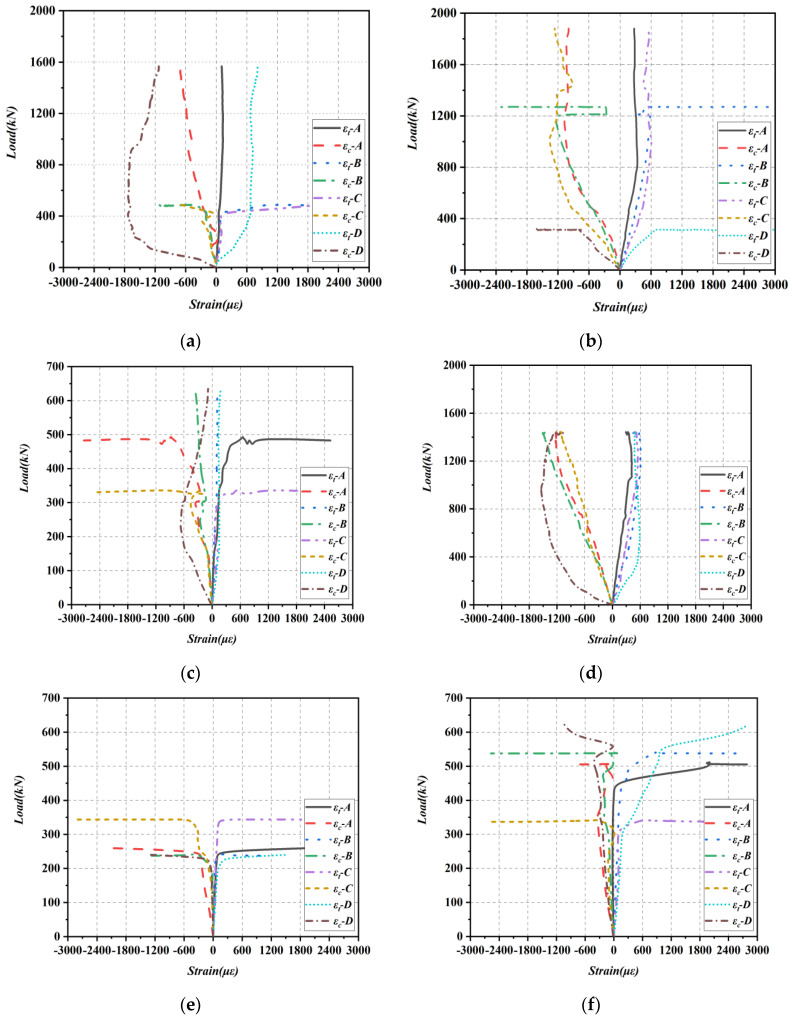

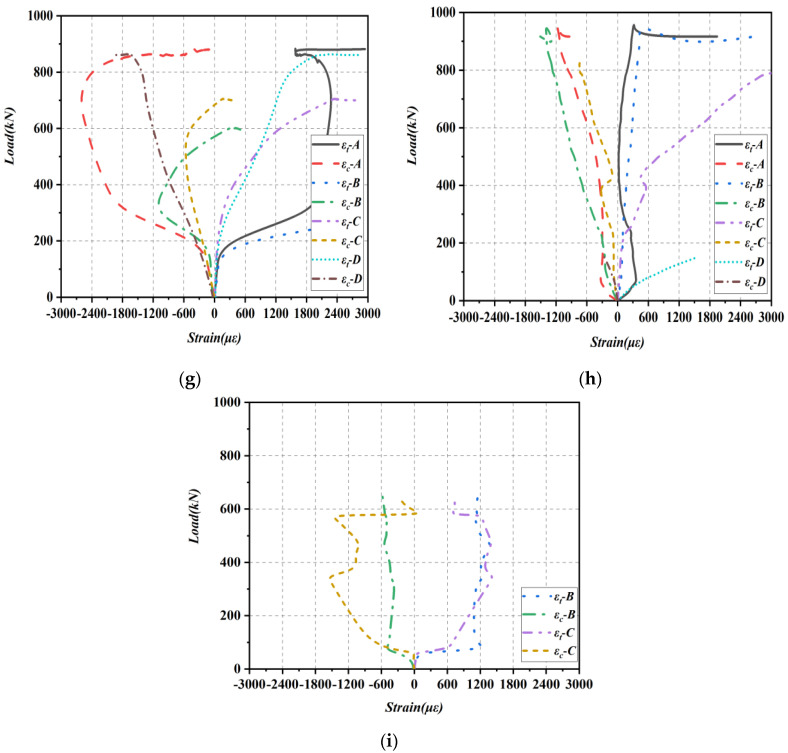
Load-principal stress relationship for concrete diagonal section. (**a**) B4; (**b**) B7; (**c**) B5; (**d**) B8; (**e**) B6; (**f**) B9; (**g**) B1; (**h**) B2; (**i**) B3.

The load-principal strain relationships for all beams at different measured points on the diagonal section are presented in Figure 10. The strain gauges on the concrete were susceptible to damage once cracks passed over them. In particular, strain gauge groups A and B of specimen B3 were unintentionally damaged at the start of the test. As a result, any data obtained after these incidents occurred were not taken into account. Prior to the cracks passing over these strain groups, the principal strains in the shear-bending section exhibited a linear increase with load. The principal strains demonstrated a gradual increase from the loading point toward the support. Notably, specimens B1, B4, B5, B7, and B8, which had smaller λ, exhibited more pronounced variations in the principal compressive strains near the support. This indicates that the concrete near the support experienced significant compressive stresses. In general, the principal tensile strains in the web section of the beam increased suddenly among the four groups of measured principal strains. This phenomenon co-occurred with the initial formation of diagonal cracks in the web section of the beam. As the diagonal cracks continued to appear, the stresses in the shear-bending section were redistributed, resulting in an irregular strain development pattern afterward.

### 3.4. Post-Cracking Shear Resistance

Equation (5) can be used to determine the post-cracking shear resistance (*PCSR*) of beams, which indicates their ability to endure loads after the initial development of the first shear diagonal crack.

(5) $$PCSR=\frac{V_{u}-V_{ci}}{V_{u}}\times 100\%$$

where $V_{u}$ is the peak shear load, taking the value of one-half of the ultimate load; $V_{ci}$ represents half of the load causing the initial shear diagonal crack to emerge.

To calculate the shear stress (ν) of the shear-bending section, the shear load (V), the effective depth of the beam (d), and web thickness (b) were used in the following equation:

(6) ν=V/bd

The ultimate shear strength, which is the maximum shear stress that a beam can withstand, was determined using the peak shear load ($V_{u}$).

Table 5 lists the calculated results for all the beams. The average value of PCSR for SR−NC beams, NSR−UHPC−0 beams, and NSR−UHPC−2 beams were 64.2%, 62.3%, and 64.4%, respectively. NSR−UHPC−2 beams exhibited comparable post-cracking shear resistance.

### 3.5. The Ultimate Shear Strengths

**Figure 11:**
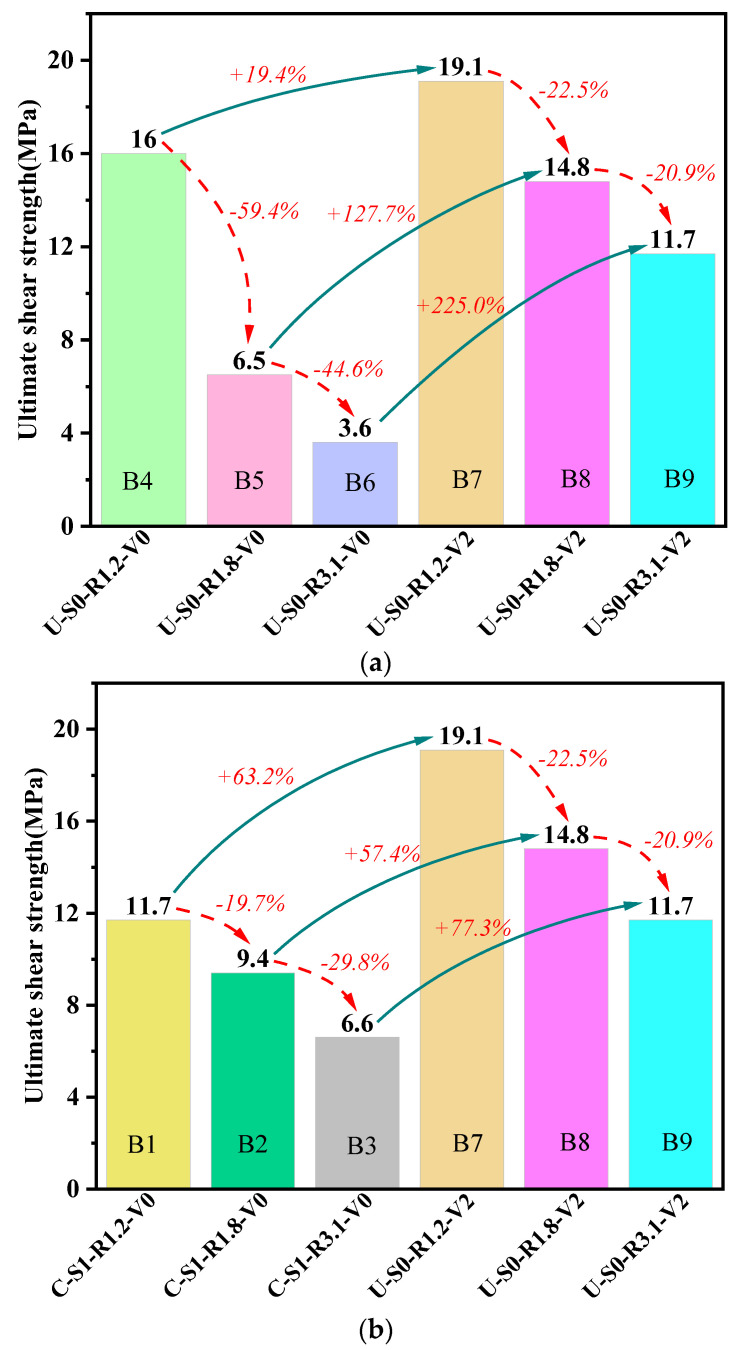
Effect experimental variables on ultimate shear strength. (**a**) NSR−UHPC−0 beams vs. NSR−UHPC−2 beams; (**b**) SR−NC beams vs. NSR−UHPC−2 beams.

The ultimate shear strengths of all tested beams are compared in Figure 11, with the dashed curved arrow lines denoting the variation of the ultimate strength across various λ. It can be seen from the figure that the ultimate shear strength decreased with the increase in the shear span-depth ratio. With an increasing λ, the ultimate shear strength of NSR−UHPC−0 beams decreased by over 40%, while the ultimate shear strength of NSR−UHPC−2 beams and SR−NC beams decreased by about 20% to 30%. It can be inferred that the λ is a significant factor that influences the shear capacity of the beams. With a decreasing λ, the diagonal shear crack had a steeper angle, thus causing an increase in the vertical component of the concrete strut.

Figure 11a depicts the influence of steel fibers on the ultimate shear strength, with the solid arrow lines indicating the comparison between different cases. The ultimate shear strengths of NSR−UHPC−2 beams (B7, B8, and B9) were increased by 19.4%, 127.7%, and 225.0%, respectively, compared to NSR−UHPC−0 beams (B4, B5 and B6). Hence, the incorporation of steel fibers in the UHPC matrix can remarkably enhance the ultimate shear strength of UHPC beams by improving the mechanical properties of the UHPC matrix and creating bridging links to bear the tensile stress in diagonal shear cracks.

Figure 11b shows the ultimate shear strength comparison between NSR−UHPC−2 beams and SR−NC beams, indicated by solid arrow lines. The ultimate shear strength of specimens B7, B8, and B9 increased by 63.2%, 57.4%, and 77.3% compared to specimens B1, B2, and B3, respectively. Consequently, NSR−UHPC−2 beams demonstrated significantly higher ultimate shear strength than SR−NC beams, indicating the feasibility and rationality of eliminating stirrups in UHPC beams, given the outstanding tensile properties of the UHPC matrix.

## 4. Shear Design Recommendations for UHPC Beams

### 4.1. French Standard Formulae

In accordance with French Standard NF P 18-710-2016 [52], the shear load capacity of UHPC beams is determined by three constituent factors, namely, the contributions from the UHPC matrix $V_{c}$, the stirrups $V_{s}$, and the steel fibers $V_{f}$. Accordingly, the expression for the shear load capacity $V_{u1}$ is:

(7) $$V_{u1}=V_{c}+V_{s}+V_{f}$$

The shear load capacity is determined in part by the properties of the UHPC matrix $V_{c}$, which can be described as:

(8) $$V_{c}=\frac{0.21}{\gamma_{\mathrm{cf}}\gamma_{E}}k_{1}f_{\mathrm{cu}}^{\frac{1}{2}}bd$$

where the comprehensive safety factor $\gamma_{\mathrm{cf}}\gamma_{E}$ is equal to 1.0 here. The prestressing improvement factor $k_{1}$ is equal to 1.0 because no prestressing was applied to tested beams in this study. The variables $f_{\mathrm{cu}}$, *b*, and *d*, respectively, denote the compressive strength of UHPC, the web thickness, and the effective depth.

The shear load capacity by stirrups $V_{s}$ is computed from the following equation:

(9) $$V_{s}=\frac{A_{\mathrm{sv}}}{s}zf_{y}\cot\theta$$

where $A_{\mathrm{sv}}$ represents the cross-sectional area of the stirrups used. The variable *s* represents the spacing of the stirrups. The variable *z* is calculated as 0.9*d* to determine the lever arm of internal forces. $f_{y}$ represents the yielding strength of the stirrup. θ represents the angle between the beam axis and the critical shear diagonal crack, taken as 45°. Non-stirrup UHPC beams are assumed to have zero shear contribution from stirrups.

The equation for calculating the steel fiber contribution $V_{f}$ is as follows:

(10) $$V_{f}=A_{b}\sigma_{f1}\cot\theta$$

where $A_{b}$ represents the effective cross-sectional area of the beam and takes the value of *bz* in this paper. $\sigma_{f1}$ represents the post-cracking strength with the relevant values listed in Table 3.

### 4.2. PCI-2021 Formulae

In accordance with the PCI-2021 report [53], the shear load capacity of UHPC beams can be segregated into three separate resistive mechanisms, namely, the tensile strength of UHPC $V_{\mathrm{cf}}$, the contribution of effective prestressing force $V_{p}$ acting in the direction of applied shear, and the resistance offered by shear reinforcement $V_{s}$. Therefore, the shear load capacity $V_{u2}$ is obtained as follows:

(11) $$V_{u2}=V_{\mathrm{cf}}+V_{s}+V_{p}$$

(12) $$V_{\mathrm{cf}}=(\frac{4f_{\mathrm{rr}}}{3})bz\cot\theta$$

(13) $$V_{s}=\frac{A_{\mathrm{sv}}f_{y}z\cot\theta }{s}$$

(14) $$\theta =29{}^{\circ}+3500\varepsilon_{s}$$

where the values for longitudinal strain $\varepsilon_{s}$ confinement are limited to under −0.40 × 10^−3^ compressive stress and 6.0 × 10^−3^ under tensile stress, which, respectively, correspond to an angle of 27.6°and 50.0°, and θ=45° is taken in this paper. $f_{\mathrm{rr}}$ is the residual tensile strength, which is listed in Table 3. In this paper, there were no prestressing tendons and stirrups in the test UHPC beam, so $V_{p}$ and $V_{s}$ was taken as zero.

### 4.3. Xu’s Formulae

Accounting for the influence of prestressing force, steel fibers, and shear span-to-depth ratio, Xu’s formulae [54] are empirical equations derived through regression analysis of a substantial number of test results for the shear load capacity of UHPC beams. Hence, the shear load capacity $V_{u3}$ of UHPC beams is given by:

(15) $$V_{u3}=V_{c}+V_{s}+V_{f}$$

(16) $$V_{c}=k_{2}\left(\frac{2}{\lambda -0.7}-0.8\right)\sqrt{f_{c}}b$$

(17) $$V_{s}=\left(0.18+0.35\lambda \right)\rho_{s}f_{y}bd$$

(18) $$V_{f}=\left(0.99-0.12\lambda \right)\lambda f_{t}bd$$

(19) $$f_{t}=0.0353f_{c}$$

where $\rho_{s}$ is stirrup ratio. When λ<1.5, λ takes 1.5, and when λ>3.0, λ takes 3.0. $k_{2}$ represents the prestressing enhancement factor. For UHPC beams that are not prestressed, the $k_{2}$ equals 1.0, whereas for prestressed UHPC beams, its value is 1.25. Given the absence of stirrup reinforcement in UHPC beams, the corresponding shear contribution from the stirrups is assumed to be negligible.

### 4.4. Comparison of Calculation Results

Table 6 lists the calculated values of shear capacity V_u1_ (French Standard formulae), V_u2_ (PCI-2021 formulae), V_u3_ (Xu’s formulae), and the corresponding ratios V_u1_/V_u,test_, V_u2_/V_u,test_, and V_u3_/V_u,test_ of the calculated shear capacity to the experimental shear capacity.

For the French standard formulae, the ratio V_u1_/V_u,test_ ranged from 0.15 to 0.90, with an average of 0.55, an STDEV of 0.24, and a coefficient of variation of 0.44. However, since the calculation formulae do not account for the important factor of λ, the coefficients in the formulae are certainly conservative for UHPC beams with λ of 1.2 and 1.8. Notably, the ratio for specimen B6 was 0.66, whereas that for specimen B9 was 0.90, indicating that the formulae were reasonably accurate for NSR−UHPC−2 beams with a λ of 3.1.

For the PCI-2021 formulae, the ratio V_u2_/V_u,test_ ranged from 0.30 to 1.35, with an average of 0.83, an STDEV of 0.33, and a coefficient of variation of 0.40. Based on the calculations, it can be observed that the PCI-2021 formulae give more accurate predictions for NSR−UHPC−2 beams than the French standard formulae because PCI-2021 formulae originated for UHPC with more than 2% steel fibers.

For the Xu’s formulae, the ratio V_u3_/V_u,test_ ranged from 0.2 to 1.62, with an average of 1.08, an STDEV of 0.45, and a coefficient of variation of 0.42. Compared with the French standard formulae and PCI-2021 formulae, the calculation of Xu’s formulae was comparable and accurate, with an average value closer to one, but the STDEV and CV were higher. This is possibly due to Xu’s formulas incorporating the effect of λ. To adjust for accuracy, this paper recommends multiplying Xu’s formulae for NSR−UHPC−2 beams by a reduction factor of 0.72.

The comparison of calculated values obtained from the three formulae indicates that the French standard formulae and PCI-2021 formulae are suitable for the shear design of NSR−UHPC−2 beams. Xu’s formulae are suitable for all UHPC beams in this paper when a reduction factor is taken into account. It is suggested that the formulae for shear capacity incorporate the factor associated with λ.

## 5. Conclusions

The shear behavior of both non-stirrup UHPC beams and stirrup-reinforced NC beams, when subjected to a three-point loading, was researched in this study, and the results led to the following conclusions.

(1) The failure modes of all nine test beams were shear failures. For SR−NC beams, specimen C-S1-R1.2-V0 failed by diagonal compression, while specimens C-S1-R1.8-V0 and C-S1-R3.1-V0 exhibited shear compression failure. Non-stirrup UHPC beams primarily failed due to shear compression, with the only exception being specimen U-S0-R3.1-V0, which failed in diagonal tensile mode. Steel fibers are a crucial factor affecting the failure mode of non-stirrup UHPC beams and can effectively improve the cracking strength of the beams. Steel fibers play a critical role in influencing the failure mode of non-stirrup UHPC beams and can effectively enhance the cracking strength of the beams.
(2) The stiffness of a beam, which is reflected in the slope of its elastic stage, can be impacted by the λ. The elastic-plastic stage of the curve is impacted by steel fibers, leading to improved ductility of the beam up to a maximum ductility coefficient of 2.75.
(3) The longitudinal reinforcements were fully utilized in NSR−UHPC−2 beams bearing shear, which had yielded before reaching ultimate shear strength. The principal strains in the shear-bending section increased linearly with the increase in the load before the cracks crossed strain rosettes and increased from the loading point toward the support, where the principal compressive strains near the support varied more significantly.
(4) The increase in the λ resulted in a decrease in ultimate shear strength, with NSR−UHPC−0 beams experiencing a reduction of over 40% in ultimate shear strength as this ratio increased. At the same time, the decrease in ultimate shear strength for both NSR−UHPC−2 beams and SR−NC beams was between 20% to 30% as the λ increased.
(5) Compared with NSR−UHPC−0 beams (B4, B5 and B6), the ultimate shear strength of NSR−UHPC−2 beams (B7, B8 and B9) increased by 19.4%, 127.7% and 225.0%, respectively. NSR−UHPC−2 beams demonstrated excellent post-cracking shear resistance and ultimate shear strength. Hence, it was considered feasible to eliminate stirrups in UHPC beams with sufficient shear capacity.
(6) French standard formulae and the PCI-2021 formulae can be used for shear design NSR−UHPC−2 beams. Xu’s formulae are suitable for all UHPC beams in this paper when a reduction factor is taken into account. The accuracy and applicability of the reduction factor need to be verified by more tests.

## Figures and Tables

**Table 1:** Specimen nomenclature and experimental parameters.

NO.	Specimens	Series	Concrete Type	Stirrup Ratio	*l* (mm)	*a* (mm)	λ	Volume Content of Steel Fiber
B1	C-S1-R1.2-V0	SR−NCbeams	C40	0.5%	800	300	1.2	0
B2	C-S1-R1.8-V0		C40	0.5%	1100	450	1.8	0
B3	C-S1-R3.1-V0		C40	0.5%	1700	750	3.1	0
B4	U-S0-R1.2-V0	NSR−UHPC−0 beams	UHPC	0	800	300	1.2	0
B5	U-S0-R1.8-V0		UHPC	0	1100	450	1.8	0
B6	U-S0-R3.1-V0		UHPC	0	1700	750	3.1	0
B7	U-S0-R1.2-V2	NSR−UHPC−2 beams	UHPC	0	800	300	1.2	2.0%
B8	U-S0-R1.8-V2		UHPC	0	1100	450	1.8	2.0%
B9	U-S0-R3.1-V2		UHPC	0	1700	750	3.1	2.0%

**Table 2:** Mix proportions of UHPC and C40 (kg/m^3^).

Component	Cement	Silica Fume	Nano-CaCO_3_	Quartz Sand	Steel Fiber	Water	Water Reducer	Stone
UHPC-0	1000	209	118	1085	0	176	53	0
UHPC-2	1000	209	118	1085	156	176	53	0
C40	368	0	0	640	0	217	0	1138

**Table 3:** Basic mechanical properties of UHPC materials.

Concrete Type	Volume Content of Steel Fiber	*f*_cu_ (MPa)	*f*_c_ (MPa)	$\sigma_{f1}$ (MPa)	$\sigma_{f2}$ (MPa)	*f*_fu_ (MPa)	$f_{\mathrm{rr}}$ (MPa)
UHPC-0	0%	126.1	106.3	3.2	2.0	10.7	4.0
UHPC-2	2%	158.8	142.0	8.7	5.4	28.1	10.5
C40	0%	43.7	38.5	–	–	–	–

Notes: $f_{\mathrm{cu}}$ = Cubic compressive strength; $f_{c}$ = Axial compressive strength; $\sigma_{f1}$ = Tested post-cracking strength; $\sigma_{f2}$ = Post-cracking strength for design; $f_{\mathrm{fu}}$ = Ultimate flexural strength; $f_{\mathrm{rr}}$ = Post-cracking residual strength.

**Table 4:** Mechanical properties of reinforcing steel.

Specimens	Reinforcing Steel Type	Diameter (mm)	Yield Strength (MPa)	Ultimate Strength (MPa)
Stirrups	HRB400	8	412.0	621.1
Longitudinal reinforcements	HRB400	25	425.1	634.3

**Table 5:** Summary of test results.

NO.	$P_{\mathrm{cr}}$ (kN)	$\sigma_{\mathrm{cr}}$ (MPa)	$P_{\mathrm{ci}}$ (kN)	$\nu_{\mathrm{ci}}$ (MPa)	$P_{u}$ (kN)	$\nu_{u}$ (MPa)	$V_{u}$ (kN)	$\Delta_{u}$ (mm)	$P_{failure}$ (kN)	$\Delta_{failure}$ (mm)	$P_{y}$ (kN)	$\Delta_{y}$ (mm)	$\mu_{\Delta }$	PCSR	Failure Pattern
B1	200	10.0	440	4.5	1146	11.7	573.0	4.00	879	6.00	1119	3.52	1.70	61.6%	DC
B2	70	5.3	320	3.3	915	9.4	457.5	4.34	775	7.67	863	4.18	1.84	65.0%	SC
B3	60	6.0	220	2.2	648	6.6	324.0	9.21	519	12.69	614	8.14	1.56	66.1%	SC
B4	118	6.0	291	3.0	1566	16.0	783.0	2.89	1240	3.08	1526	2.81	1.10	81.4%	SC
B5	76	5.7	235	2.4	634	6.5	317.0	3.14	516	3.81	579	2.91	1.31	62.9%	SC
B6	58	5.8	200	2.0	349	3.6	174.5	5.13	292	5.61	300	3.35	1.67	42.7%	DT
B7	220	11.0	549	5.6	1871	19.1	935.5	4.13	1487	6.85	1772	3.98	1.72	70.7%	SC
B8	157	11.8	510	5.2	1446	14.8	723.0	6.50	971	8.92	1353	4.53	1.97	64.7%	SC
B9	139	13.9	479	4.9	1141	11.7	570.5	17.43	288	24.68	1048	8.98	2.75	58.0%	SC

Notes: $P_{\mathrm{cr}}$ = Load of initial flexural crack; $\sigma_{\mathrm{cr}}$ = Flexural cracking strength; $P_{\mathrm{ci}}$ = Load of initial diagonal crack; $\nu_{\mathrm{ci}}$ = Diagonal cracking strength; $P_{u}$ = Ultimate load; $V_{u}$ = Peak shear load; The ultimate shear strength ($\nu_{u}$) is given by: $\nu_{u}=V_{u}/\mathrm{bd}$, where, b is web thickness and d is effective depth of the beam; $\Delta_{u}$ = Mid-span deflection of ultimate load; PCSR denotes the post diagonal cracking shear resistance; $P_{\mathrm{failure}}$ = Failure load; $\Delta_{\mathrm{failure}}$ = Mid-span deflection of failure load; $P_{y}$ = Yield load; $\Delta_{y}$ = Mid-span deflection of yield load; The ductility coefficients ($\mu_{\Delta }$) is given by: $\mu_{\Delta }=\Delta_{failure}$/$\Delta_{y}$.

**Table 6:** Comparison between experimental results and calculated ones.

Experimental Results		French Standard Formulae					PCI-2021 Formulae				Xu’s Formulae				
NO.	$V_{u,test}$	$V_{c}$	$V_{s}$	$V_{f}$	$V_{u1}$	$V_{u1}/V_{u,test}$	$V_{cf}$	$V_{s}$	$V_{u2}$	$V_{u2}/V_{u,test}$	$V_{c}$	$V_{s}$	$V_{f}$	$V_{u3}$	$V_{u3}/V_{u,test}$
B4	783.0	115.3	0	0	115.3	0.15	234.7	0	234.7	0.30	857.1	0	0	857.1	1.09
B5	317.0	115.3	0	0	115.3	0.36	234.7	0	234.7	0.74	513.3	0	0	513.3	1.62
B6	174.5	115.3	0	0	115.3	0.66	234.7	0	234.7	1.35	35.1	0	0	35.1	0.20
B7	935.5	129.4	0	382.9	512.3	0.55	616.1	0	616.1	0.66	990.6	0	297.8	1288.4	1.38
B8	723.0	129.4	0	382.9	512.3	0.71	616.1	0	616.1	0.85	593.3	0	341.5	934.8	1.29
B9	570.5	129.4	0	382.9	512.3	0.90	616.1	0	616.1	1.08	40.5	0	463.3	503.8	0.88
		Mean of $V_{u1}/V_{u,test}$:0.55; STDEV:0.24; CV:0.44.					Mean of $V_{u2}/V_{u,test}$:0.83; STDEV:0.33; CV:0.40.				Mean of $V_{u3}/V_{u,test}$:1.08; STDEV:0.45; CV:0.42.				

## Data Availability

Not applicable.
